# Androgens Upregulate Cdc25C Protein by Inhibiting Its Proteasomal and Lysosomal Degradation Pathways

**DOI:** 10.1371/journal.pone.0061934

**Published:** 2013-04-18

**Authors:** Yu-Wei Chou, Li Zhang, Sakthivel Muniyan, Humera Ahmad, Satyendra Kumar, Syed Mahfuzul Alam, Ming-Fong Lin

**Affiliations:** 1 Department of Biochemistry and Molecular Biology, College of Medicine, University of Nebraska Medical Center, Omaha, Nebraska, United States of America; 2 Beijing Friendship Hospital affiliated to the Capital Medical University, Beijing Digestive Disease Center, Beijing, China; 3 Eppley Institute for Research in Cancer and Allied Diseases, University of Nebraska Medical Center, Omaha, Nebraska, United States of America; 4 Department of Surgery/Urology, University of Nebraska Medical Center, Omaha, Nebraska, United States of America; 5 College of Pharmacy, Kaohsiung Medical University, Kaohsiung, Taiwan, ROC; The Chinese University of Hong Kong, Hong Kong

## Abstract

Cdc25C is a cell cycle protein of the dual specificity phosphatase family essential for activating the cdk1/Cyclin B1 complex in cells entering into mitosis. Since altered cell cycle is a hallmark of human cancers, we investigated androgen regulation of Cdc25C protein in human prostate cancer (PCa) cells, including androgen-sensitive (AS) LNCaP C-33 cells and androgen-independent (AI) LNCaP C-81 as well as PC-3 cells. In the regular culture condition containing fetal bovine serum (FBS), Cdc25C protein levels were similar in these PCa cells. In a steroid-reduced condition, Cdc25C protein was greatly decreased in AS C-33 cells but not AI C-81 or PC-3 cells. In androgen-treated C-33 cells, the Cdc25C protein level was greatly elevated, following a dose- and a time-dependent manner, correlating with increased cell proliferation. This androgen effect was blocked by Casodex, an androgen receptor blocker. Nevertheless, epidermal growth factor (EGF), a growth stimulator of PCa cells, could only increase Cdc25C protein level by about 1.5-fold. Altered expression of Cdc25C in C-33 cells and PC-3 cells by cDNA and/or shRNA transfection is associated with the corresponding changes of cell growth and Cyclin B1 protein level. Actinomycin D and cycloheximide could only partially block androgen-induced Cdc25C protein level. Treatments with both proteasomal and lysosomal inhibitors resulted in elevated Cdc25C protein levels. Immunoprecipitation revealed that androgens reduced the ubiquitination of Cdc25C proteins. These results show for the first time that Cdc25C protein plays a role in regulating PCa cell growth, and androgen treatments, but not EGF, greatly increase Cdc25C protein levels in AS PCa cells, which is in part by decreasing its degradation. These results can lead to advanced PCa therapy via up-regulating the degradation pathways of Cdc25C protein.

## Introduction

Cell cycle progression is controlled by the sequential activation of cyclin-dependent kinase (CDK) whose activities are tightly regulated by cyclins, CDK inhibitor, and a variety of other proteins [1], [2]. Cell division cycle (Cdc) 25 proteins are highly conserved dual specificity phosphatases that activate CDK complexes, which in turn regulate the progression through different phases of cell cycle [3]. Cdc25 proteins are encoded by a multigene family, consisting of three isoforms with different molecular weights: Cdc25A, Cdc25B and Cdc25C [4], [5], [6]. Although it was initially proposed that each Cdc25 has a specific role in a particular stage of the cell cycle, including results from mutant mice experiments [7], [8], [9]; current results indicate that these Cdc25 proteins have overlapping functions [3].

Cdc25A is involved in mitosis and the checkpoint signaling pathway [10], and also functions as an oncogenic protein with overexpression in several human malignancies including liver, breast and ovarian cancers [11]. Cdc25B plays a role in S- and G2-phases and activates Cdc2/cyclin B at mitotic entry [10]. Results of several studies show the importance of Cdc25C in cell cycle regulation during the G2-to-mitosis transition [12], [13], [14], [15], [16], [17] and in response to DNA damage and replicational stress [18], [19], [20]. Upon DNA damage, cells will arrest the cell cycle and induce the transcription of genes needed for DNA repair. Cdc25C can be negatively regulated by Ser-216 phosphorylation for cytoplasmic sequestration [19], [21]. Cdc25C activity can also be inhibited via phosphorylation by checkpoint kinases Chk1 and Chk2 when there is a DNA damage, which will prevent cyclin B/cdk1 activation [22]. Activated Chk kinases phosphorylate Cdc25C at Ser-216, blocking the activation of cdk1 and subsequent transition into the M phase [23]. Additionally, Cdc25C can be inactivated by Wee1 and Myt1 kinases in the cyclin B/cdk1 complex [24].

Due to the importance of Cdc25 members in cell cycle regulation, this group of enzymes has received much attention. However, the majority of studies on Cdc25 members thus far have been focused on investigating the phosphorylation and consequent subcellular localization and cell cycle regulation. Very little data is available regarding the activator of Cdc25 members, especially Cdc25C and its biological significance relating to specific carcinogenesis [25].

In this study, we investigated the regulation of protein tyrosine phosphatase (PTP) proteins by androgens in prostate cancer (PCa) cells because androgens play a critical role in diverse activities of prostate cells including normal development, differentiation and pathogenesis. Androgen sensitivity is also a hallmark of PCa. To study androgen effect on PCa cell proliferation, we analyzed the protein level of cellular prostatic acid phosphatase (cPAcP), an authentic PTP, as a marker for androgen action; because cPAcP functions as a negative growth regulator by dephosphorylating ErbB-2 tyrosine phosphorylation [26], [27], [28]. In growth-stimulated PCa cells by both androgen and EGF, the cPAcP level is decreased [29], [30]. Our data clearly showed that the Cdc25C protein level is positively correlated with androgen status and plays a role in regulating PCa cell proliferation. In androgen-treated cells, cPAcP is decreased and Cdc25C is up-regulated, leading to growth stimulation. Despite the fact that there are many studies on Cdc25C, to the best of our knowledge, this is the first report that clearly showed Cdc25C protein is up-regulated by androgens, but not by EGF, and plays a critical role in regulating both basal and androgen-stimulated PCa cell growth. Furthermore, androgens up-regulate Cdc25C protein levels at least in part by inhibiting its degradation pathways, which lead to PCa cell proliferation. Our results may lead to the development of effective therapy toward advanced castration-resistant PCa via down-regulating Cdc25C protein levels.

## Materials and Methods

### Materials

Fetal bovine serum (FBS), RPMI 1640 culture medium, glutamine and gentamicin were purchased from Invitrogen (Carlsbad, CA, USA). Charcoal/dextran-treated, certified FBS, anti-β-actin antibody (Ab) and 5α-dihydrotestosterone (DHT) were obtained from Sigma (St. Louis, MO, USA). Polyclonal Ab to Cdc25C protein (Sc327) was purchased from Santa Cruz Biotechnology, Inc. (Santa Cruz, CA, USA). Rabbit anti-PAcP ATM-3 antisera were obtained as described previously [29]. The Cdc25C shRNA plasmid and control plasmid, and the horseradish peroxidase-conjugated anti-rabbit and anti-mouse immunoglobulin G (IgG) were purchased from Santa Cruz Biotechnology, Inc. (Santa Cruz, CA, USA). The Cdc25C cDNA plasmid was purchased from OriGene Technologies, Inc. (Rockville, MD, USA). All other chemicals were as described previously [26], [31], [32], [33].

### Cell culture

Human prostate carcinoma cell lines including LNCaP cells [34], MDA PCa2b cells [35], PC-3 cells [36], DU 145 cells [37] and VCaP cells [38] were originally purchased from the American Type Culture Collection (Rockville, MD, USA). LNCaP, PC-3 and DU 145 cells were routinely maintained in the regular culture medium, i.e., phenol red-positive RPMI 1640 medium supplemented with 5% FBS, 2 mM glutamine and 50 µg/mL gentamicin [31]. MDA PCa2b cells were cultured in BRFF-HPC1 medium containing 20% FBS, 2 mM glutamine and 50 µg/mL gentamicin [27], [39]; while VCaP cells were cultured in DMEM containing 10% FBS, 2 mM glutamine, 50 µg/mL gentamicin and 10 nM DHT [40]. Cells were split once per week, which was defined as one passage. LNCaP cells with passage numbers less than 33 were designated as C-33, those with numbers greater than 80 as C-81 [26], [41]. LNCaP C-33 cells express functional androgen receptor (AR), are androgen-sensitive (AS) cells, and cell growth is greatly decreased in the absence of androgen. Despite C-81 cells expressing a similar level of functional AR to C-33 cells, they are androgen-independent (AI), -responsive cells [26], [41]. Thus, C-81 cells grow very well in the absence of androgen with a low degree of androgen stimulation, mimicking advanced clinical PCa [26], [41].

For DHT treatments, LNCaP C-33 and C-81 cells were steroid-starved for 48 hr in a steroid-reduced (SR) medium, i.e., phenol red-free RPMI 1640 medium containing 5% charcoal/dextran-treated, heat-inactivated certified FBS, 2 mM glutamine and 50 µg/mL gentamicin. Cells were then exposed to 10 nM DHT for a time period specified in each experiment.

### Cdc25C cDNA and shRNA plasmids transfection and cell growth determination

For Cdc25C cDNA and shRNA plasmid transfection, LNCaP C-33 and PC-3 cells were plated in regular medium at a density of 1.2×10^4^ and 1×10^4^ cells/cm^2^, respectively, for 72 hr and then transfected with Cdc25C cDNA or shRNA. Control cells were transfected with the vector alone. Five hours after transfection, all transfected cells were fed with RPMI medium with 10% FBS for 24 hr. In cDNA transfection experiments, both cDNA- and vector alone-transfected C-33 cells were maintained in fresh regular medium for 2 days and then harvested for cell number counting. In shRNA transfection experiments, shRNA- and vector alone-transfected C-33 cells were transferred to SR medium for 2 days and then treated with or without 10 nM DHT for 2 days prior to cell number counting; while shRNA-transfected PC-3 cells were maintained in regular medium for 2 days before cell counting. Cell numbers were counted in a Nexcelom Biosciences Cellometer^TM^ Auto T4 Image-based cell counter (Nexcelom Biosciences, Lawrence, MA, USA) [27]. Total cell lysate proteins were analyzed by western blotting for Cdc25A, Cdc25B, Cdc25C, cyclin B1 and cyclin D1 protein levels. β-actin was used as a loading control.

### Immunoblotting and immunoprecipitation

For immunoblotting, subconfluent cells were harvested by scraping. After centrifugation for 10 min at 1,000x g at 4°C, the pelleted cells were rinsed with ice-cold 20 mM HEPES-buffered saline, pH 7.0, and then lysed in ice-cold cell lysis buffer containing protease and phosphatase inhibitors. The detailed protocols for immunoblotting were described previously [26], [27], [31]. The intensity of hybridization band was semiquantified by densitometric analyses of autoradiograms with different exposure time periods using ImageJ.exe program (http://rsb.info.nih.gov/). To calculate the relative protein level, each densitometer reading was first normalized to that of the corresponding β-actin protein. This ratio was further compared to that of control cells, which was designated as 1.0.

For immunoprecipitation, cells were harvested and washed with ice-cold 20 mM HEPES-buffered saline, pH 7.0, pelleted by centrifugation, and lysed on ice for 20 minutes with lysis buffer (50 mM Tris, pH 7.4, 150 mM NaCl, 5 mM EDTA, 0.5% NP-40, protease inhibitor cocktail). Ab to Cdc25C protein (3 µg) was incubated with Protein A-Sepharose beads (50 µL of 10% suspension) in 500 µL of lysis buffer for 1 hour at 4°C. Cell lysates (0.3 mg) were incubated with Ab-coated Protein A-Sepharose beads in a volume of 500 µL at 4°C for 2 hr. Beads were washed three times (1 mL each) with ice-cold lysis buffer. Immunoprecipitated proteins were eluted by heating at 95°C for 5 minutes in Laemmli sample buffer (50 mM Tris HCl, pH 6.8, 2% SDS (v/v), 0.001% bromophenol blue, 10% glycerol (v/v), 100 mM dithioerythritol), and then subjected to immunoblot analyses.

### Flow cytometry

For analyzing DHT effect on PCa cell cycle, AS C-33 cells were seeded at a density of 2×10^4^ cells/cm^2^ in regular medium for 3 days followed by maintaining in SR medium for 2 days. Cells were then treated with 10 nM DHT or solvent alone. For a time period specified in each experiment, cells were trypsinized, harvested and washed twice by Hank's balanced salt solution. Cells were treated with 70% ethanol at 4°C for 1 hr, washed with PBS and spun down by centrifugation. The DNA in ethanol-fixed cells was stained by the propidium iodide (PI) staining reagent at 4°C for 30 minutes. The PI staining reagent was prepared in PBS, pH 7.4, containing 0.1% Triton X-100, 0.1 mM EDTA disodium salt, 0.05 mg/ml RNaseA (50 U/mg), and 50 mg/ml PI [42]. The determination of cell cycle distribution was carried out using a Becton-Dickinson fluorescence-activated cell sorter (FACSCalibur, Becton Dickinson, San Jose, CA, USA) at the UNMC Flow Cytometry Core Facility.

### Statistical Analysis

Each set of experiments was performed in duplicate or triplicate, specified in each figure legend or experimental design, repeated at least 2–3 times and the mean and standard error or standard deviation values of experimental results were calculated. Paired two-tailed Student's *t*-tests were used for comparison between each group. *p*<0.05 was considered statistically significant [28], [43].

## Results

### Expression profiles of protein tyrosine phosphatases in different prostate cancer cells

**Figure 1:**
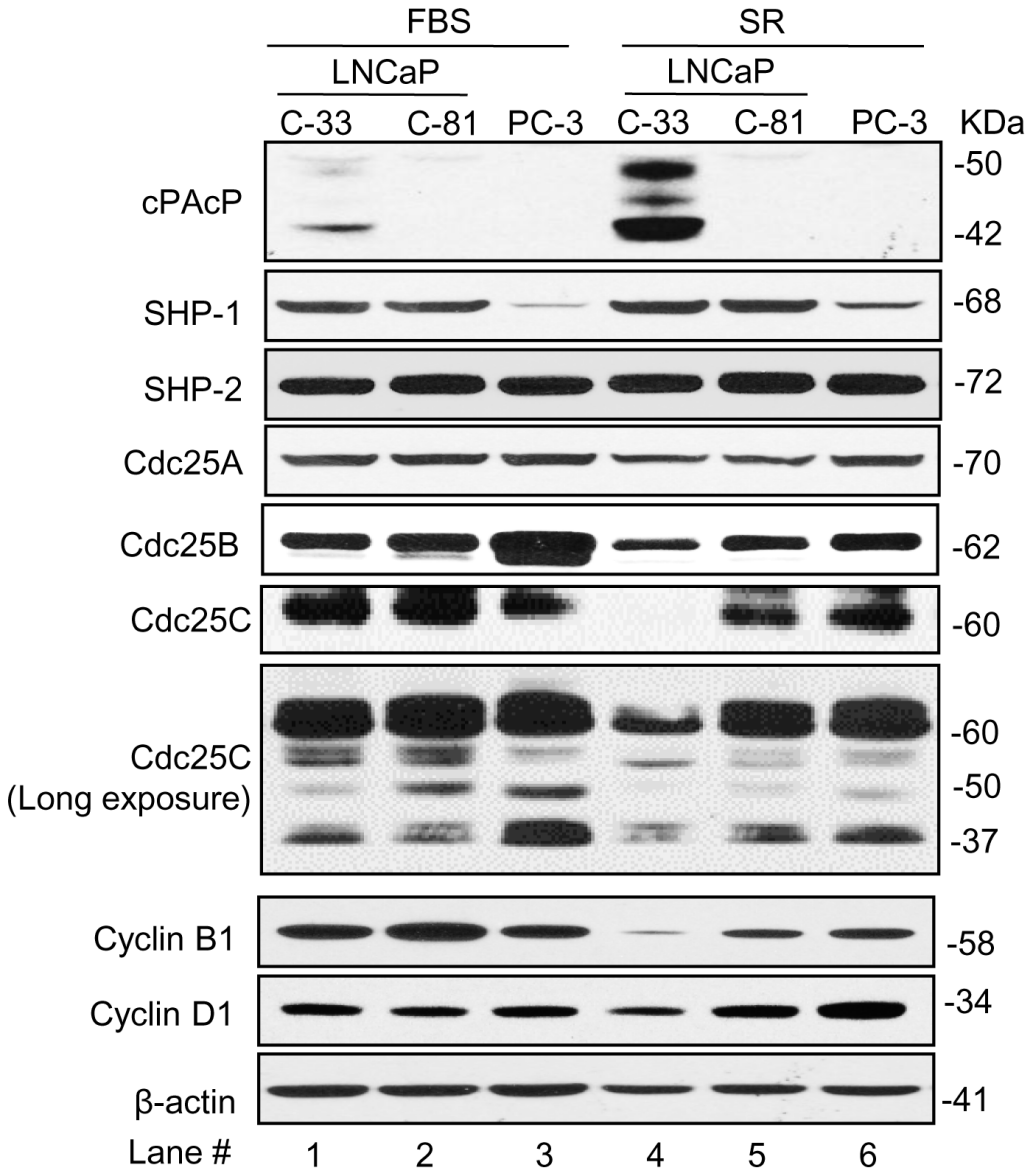
Expression profiling of protein phosphatases and cell cycle proteins in different PCa cells. LNCaP C-33, C-81 and PC-3 PCa cells were plated at a density of 8×10^3^, 6×10^3^ and 4.8×10^3^ cells/cm^2^, respectively, in duplicates for 3 days in regular medium. (Left FBS panel, lanes #1–3) Cells were replaced with fresh regular medium for 24 hr and then harvested. (Right SR panel, lanes #4–6) All three PCa cells were then steroid-starved for 48 hr in a steroid-reduced (SR) medium and then harvested. Total cell lysate proteins were analyzed for cPAcP, SHP1, SHP2, Cdc25A, Cdc25B, Cdc25C, Cyclin B1 and Cyclin D1. Cdc25C spliced forms were observed upon long exposure of films (lower panel of Cdc25C). β-actin was analyzed and used as a loading control.

We conducted analyses on the protein profiling of PTPs in PCa cells. We first validated the cell model system, including AS LNCaP C-33 and AI LNCaP C-81 as well as PC-3 cells, by analyzing the status of cPAcP because cPAcP exhibits the PTP activity and its protein level inversely correlates with PCa cell growth [26], [29], [30], [44]. In regular medium containing FBS, cPAcP is expressed in slow growing C-33 cells, while low or no expression in rapidly growing AI C-81 and PC-3 cells (Fig. 1, FBS, lanes #1–3), as we reported previously [26]. In those cells, as shown in Fig. 1 (FBS, lanes #1–3), SHP-1 and SHP-2 protein levels were very similar, except PC-3 cells had a low level of SHP-1 protein [28]. Among three Cdc25 family members, the protein level of Cdc25A was very similar among these three cell lines; while Cdc25B is higher in PC-3 cells than in C-33 and C-81 cells, and Cdc25C is slightly higher in C-81 cells than in C-33 and PC-3 cells (Fig. 1, lanes #1–3). We also analyzed the spliced forms of Cdc25C protein upon long term exposure of x-ray films to ECL reagents-treated blots. While there are some minor differences in the spliced form proteins of Cdc25C among three cell lines; similar species of spliced form proteins were detected. We semiquantified the protein level of the mature form Cdc25C protein in the pair of LNCaP cells by densitometric analyses on autoradiograms followed by calculating the ratio. The ratio of Cdc25C protein level in AI C-81 cells was about 1.2-fold of that in AS C-33 cells. Interestingly, while all cells had a similar level of Cyclin D1 protein; the Cyclin B1 protein level in C-81 cells was higher than in C-33 and PC-3 cells (Fig. 1, FBS panel, lanes #1–3).

We examined the effect of SR media on the phosphatase profiling. The SR media contain the phenol red-free medium with charcoal/dextran-treated, certified FBS in which many small molecules including steroids and growth factors were removed. In SR condition, C-33 cell growth was greatly decreased and cPAcP was elevated, higher than that in regular medium (Fig. 1, lane #4 *vs*. #1) [28]. In those three cell lines in SR media, while the protein levels of SHP-1 and SHP-2 remained essentially the same as that seen in regular medium (Fig. 1, FBS panel vs. SR panel); the protein levels of Cdc25 A and B were slightly decreased in AS LNCaP C-33 cells, lower than in AI C-81 and PC-3 cells (Fig. 1). The ratio of intensities of hybridization bands of Cdc25 A and B in C-33 cells were about 0.6- and 0.4-fold of that in AI C-81 cells, respectively, by densitometric analyses on western blots and followed by normalizing to that in AI C-81 cells. Unexpectedly, in SR conditions, the mature form Cdc25C protein level was greatly diminished in C-33 cells and could be detected only upon prolonged exposure of films (Fig. 1, Long exposure, lane #4). Among spliced form proteins, the 50 kDa spliced form protein of Cdc25C decreased in all three cell lines under SR condition (Fig. 1, Long exposure, lanes #4–6). In SR media, Cyclin B1 and D1 protein levels were also greatly decreased in AS C-33 cell, indicating a slow cell growth in SR media; while only Cyclin B1 slightly decreased in AI C-81 and PC-3 cells (Fig. 1, SR panel). The data collectively indicate that among three Cdc25 members, Cdc25C protein is the most sensitive member to the SR condition.

### Effects of androgens on Cdc25C protein level in PCa cells

Since Cdc25C protein exhibits the most distinct sensitivity to SR conditions among three Cdc25 members (Fig. 1, lane #4), and also since SR media contain reduced amounts of steroids and growth factors, we first examined DHT dosage effect on Cdc25C protein level in AS LNCaP C-33 cells, and AI LNCaP C-81 cells were used as controls. Our results showed that Cdc25C protein level was greatly elevated in C-33 cells by 10 nM and 100 nM DHT treatments for 48 hr, following a dose-dependent manner (data not shown). Cyclin B1 and D1 were also elevated, following the same fashion in those DHT-treated cells. Conversely, cPAcP protein levels decreased with increasing concentrations of DHT in C-33 cells (data not shown) as reported previously [26], [29], [31]. On the contrary, C-81 cells had high endogenous levels of Cdc25C protein in the absence of DHT, therefore, the DHT effect on Cdc25C protein levels in these cells was greatly reduced (data not shown). Similarly, DHT had only minor effects on Cyclin B1 and D1 proteins of high basal levels in C-81 cells, and in these C-81 cells, cPAcP protein level was very low and could be seen only upon prolonged exposure of films (data not shown).

**Figure 2:**
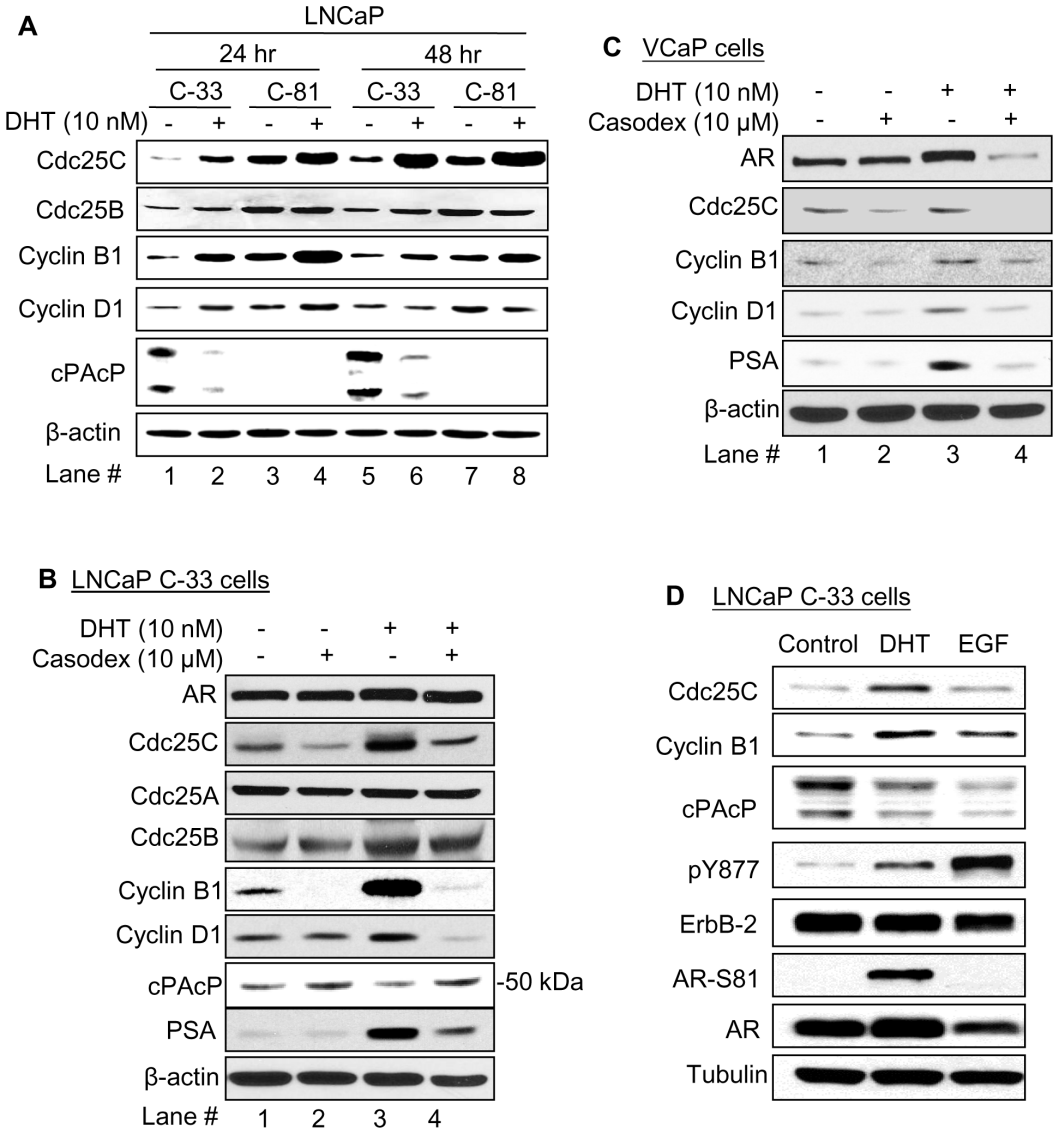
Effects of androgens on Cdc25C and cell cycle protein levels in PCa cells. (A) LNCaP C-33 and C-81 cells were seeded at a density of 8×10^3^ and 6×10^3^ cells/cm^2^, respectively, in duplicates for 3 days in regular medium. Cells were steroid-starved for 48 hr in SR medium and then treated with 10 nM DHT for 24 hr and 48 hr. Total cell lysate proteins were harvested and analyzed for Cdc25C, Cdc25B, cPAcP, Cyclin B1 and Cyclin D1 proteins. β-actin was detected for serving as a loading control. (B) AS LNCaP C-33 and (C) VCaP cells were plated at a density of 8×10^3^ and 2×10^4^ cells/cm^2^, respectively, for 3 days, and then steroid starved for 48 hr in SR medium. Cells were treated with 10 nM DHT with or without 10 µM Casodex for 48 hr. (B) For C-33 cells, total cell lysate proteins were analyzed for AR, Cdc25A, Cdc25B, Cdc25C, cPAcP, PSA, Cyclin D1 and Cyclin B1 protein levels. (C) For VCaP cells, total cell lysate proteins were analyzed for AR, Cdc25C, Cyclin D1, Cyclin B1 and PSA protein levels. β-actin was analyzed in each experiment and used as a loading control. (D) Effect of DHT *vs*. EGF on Cdc25C protein level in C-33 cells. Steroid-starved C-33 cells were treated with 10 nM DHT or 10 ng/ml EGF for 24 hr, and control cells were treated with solvent alone. All cells were harvested for analyzing Cdc25C protein level. As controls, cPAcP, ErbB-2 and its tyrosine phosphorylation at Y877, AR and its phosphorylation at S81, and Cyclin B1 were also analyzed. Tubulin was detected for serving as a loading control.

Since 10 nM DHT had the optimal effect on the Cdc25C protein level, it was used in subsequent experiments. Kinetic analyses showed that 10 nM DHT could greatly increase the Cdc25C protein level in C-33 cells upon 24 hr and 48 hr treatments (Fig. 2A, lane #2 *vs*. #1 and lane #6 *vs*. #5). In comparison, DHT has much less effect on Cdc25B protein than on Cdc25C (Fig. 2A). In those same DHT-treated C-33 cells, Cyclin B1 and D1 levels were elevated; while cPAcP was decreased in DHT-treated cells (Fig. 2A). In comparison, DHT effects on these protein levels in AI C-81 cells were greatly decreased (Fig. 2A, lane #4 *vs*. #3 and #8 *vs*. #7). Thus, DHT effect on the Cdc25C protein level in C-33 cells followed a kinetic response.

We analyzed if AR activity is required in DHT-increased Cdc25C protein. LNCaP C-33 and VCaP cells, another androgen-responsive PCa cell line, were treated with 10 nM DHT in the presence or absence of 10 µM Casodex, an AR blocker in clinical usage. When these two AS PCa cells were treated concurrently with Casodex, DHT effect on Cdc25C and/or cPAcP protein levels were blocked (Figs. 2B & 2C, lane #4 *vs*. #3). Casodex could also prevent DHT from increasing the protein levels of Cyclin B1 and Cyclin D1 (Figs. 2B & 2C). Consistently, DHT has no effect on Cdc25A with a low degree of effects on Cdc25B protein level in C-33 cells (Fig. 2B). As a control, Casodex blocked DHT-induced PSA protein levels, an androgen-regulated protein (Figs. 2B & 2C). Thus, despite that there were differential effects on AR protein levels in these two cells; a functional AR is apparently required in DHT-induced Cdc25C protein level.

Since EGF can stimulate LNCaP cell proliferation with a decrease of cPAcP activity [29], and also since the charcoal-treated FBS in SR medium also contain reduced amounts of small molecules including EGF other than steroids [29], we examined if EGF treatment could also increase Cdc25C protein levels. Unexpectedly, while Cdc25C and Cyclin B1 protein levels were greatly elevated in 24 hr DHT-treated C-33 cells; these two proteins were increased by only up to 2-fold in EGF-treated C-33 cells upon 24 hr-treatment (Fig. 2D). As controls, in those same cells, EGF treatment greatly cross-activated ErbB-2 tyrosine phosphorylation as indicated by great increase of pY877 level, and DHT treatment could activate both AR as shown by S81 phosphorylation and ErbB-2 by pY877 phosphorylation, although to a lesser degree on activating ErbB-2 than EGF (Fig 2D). Furthermore, upon both DHT and EGF treatments, cPAcP levels were decreased (Fig. 2D). Since EGF exhibits a rapid effect on signaling transduction, we analyzed EGF effect on Cdc25C protein levels with 1 hr treatment. Our results revealed that 1 hr EGF treatment had no significant effect on Cdc25C protein level in C-33 cells (data not shown). The data together clearly show that Cdc25C is the most sensitive Cdc25 member to androgens in AS PCa cells in which AR is required for this effect. Furthermore, DHT, but not EGF, can greatly increase Cdc25C protein level; while both factors can cause a decrease of cPAcP protein level and stimulate LNCaP C-33 cell proliferation (Fig. 2D) [29].

### Effect of Cdc25C protein expression on the growth of PCa cells

**Figure 3:**
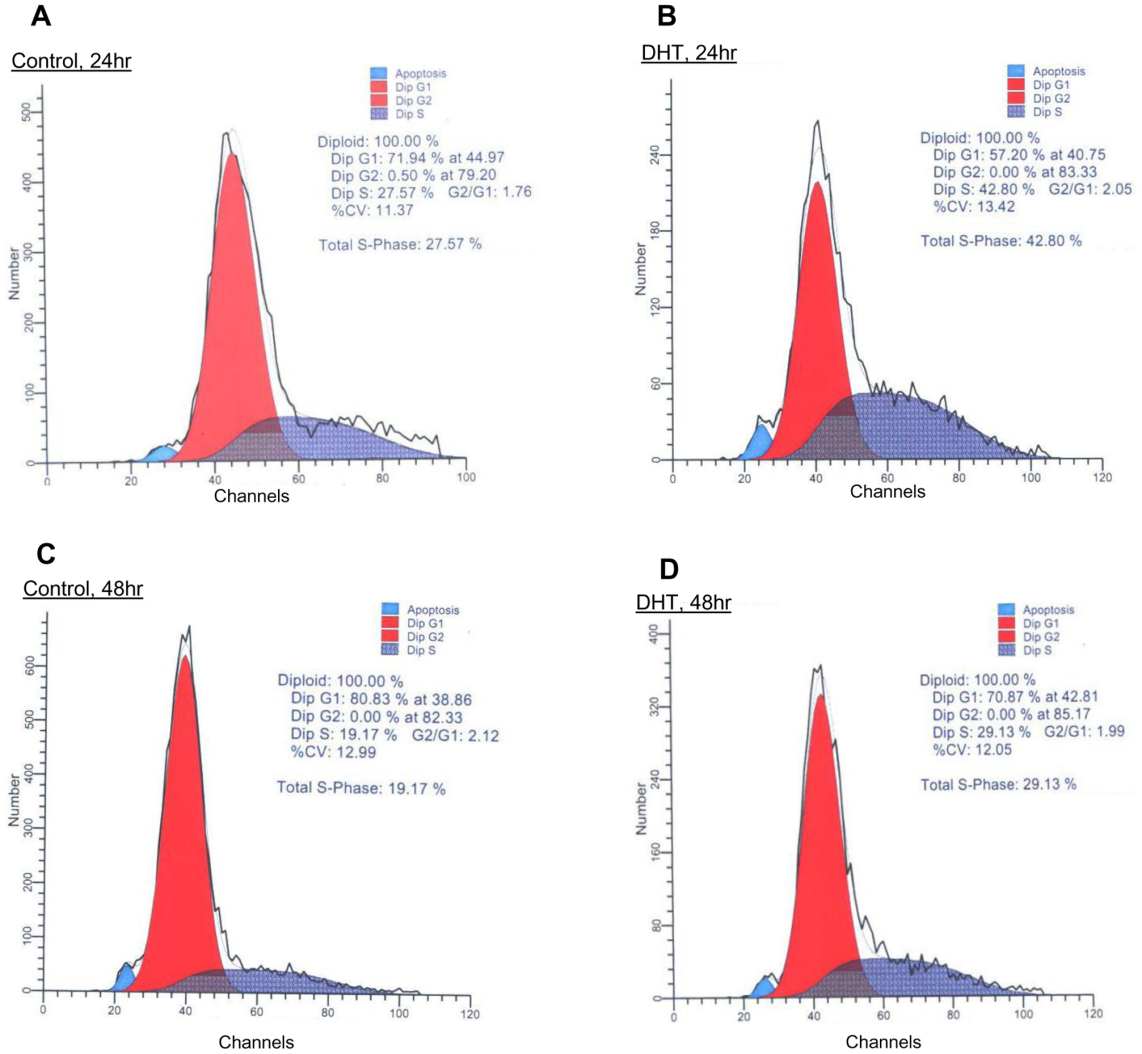
Androgen effects on the cell cycle of LNCaP C-33 cells. AS LNCaP C-33 cells were seeded at a density of 2×10^4^ cells/cm^2^ in regular medium for 3 days followed by maintaining in SR medium for 2 days. Cells were then fed with fresh SR medium and treated with 10 nM DHT or solvent alone as control cells. (A & B) One set of cells were harvested at 24 hr treatment, and (C & D) another set of cells were harvested upon 48 hr-treatment for analyzing cell cycle distribution by Flow cytometry analyses.

We investigated the biological significance of elevated Cdc25C by DHT on cell growth by performing cell cycle analysis using flow cytometry. In DHT-treated C-33 cells, Cdc25C protein level was elevated (Fig. 2); concurrently, the percentage of cell population in the S phase of cell cycle was increased (Fig. 3). In DHT-treated C-33 cells, the population of cells in the S phase was increased by approximately 50%; i.e., from 28% to 43% at 24 hr (Figs. 3A & 3B) and from 19% to 29% at 48 hr treatment (Figs. 3C & 3D). Thus, Cdc25C protein level is associated with DHT-stimulated cell proliferation, and concurrently, Cyclin B1 and D1 protein levels were also elevated (Fig. 2).

**Figure 4:**
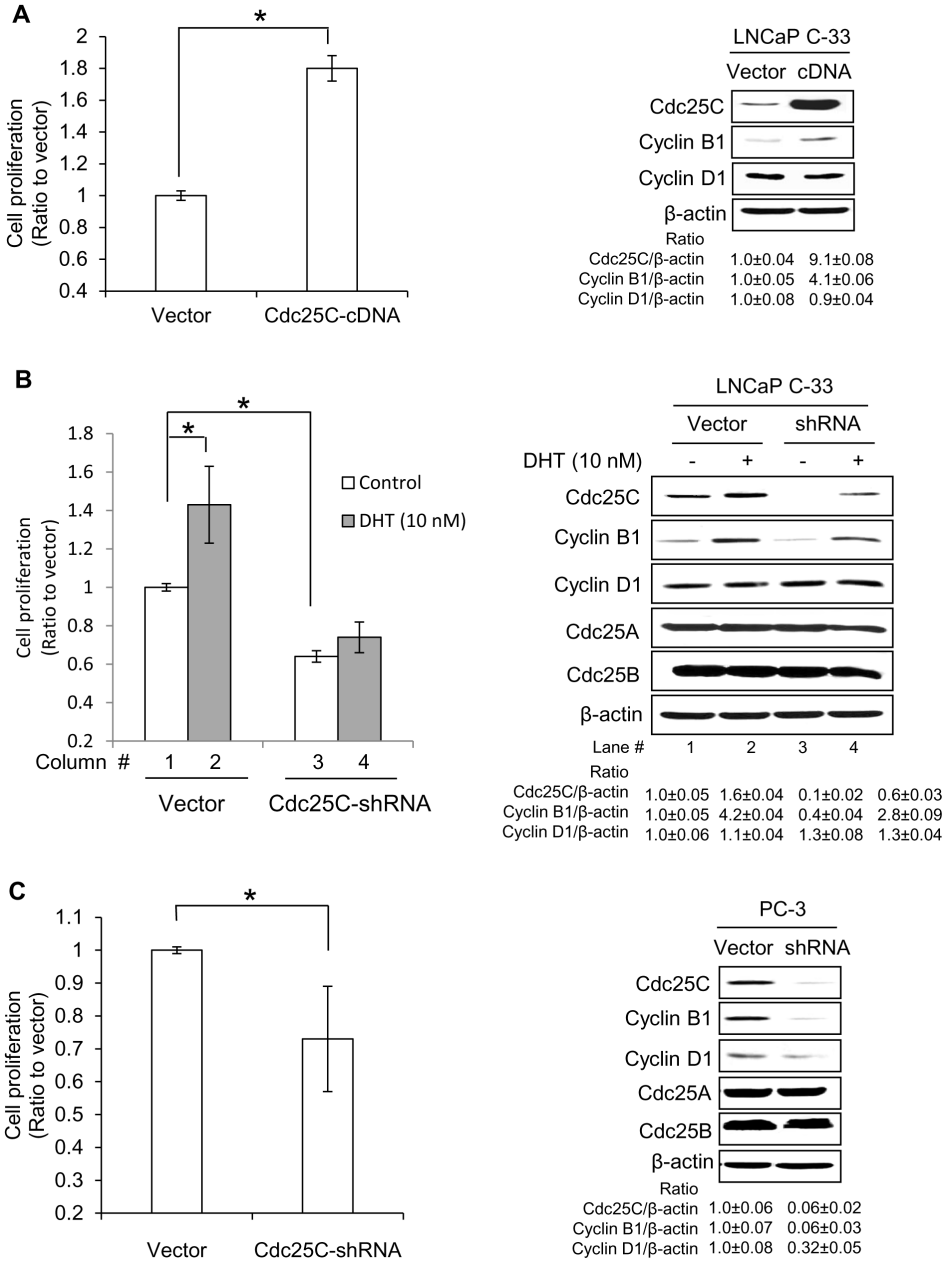
Effect of Cdc25C protein expression on the proliferation of PCa cells. (A,B) LNCaP C-33 and (C) PC-3 cells were plated at a density of 1.2×10^4^ or 1×10^4^ cells/cm^2^ for 72 hr and then transfected with Cdc25C cDNA (A) or shRNA (B,C) plasmids. Control cells were transfected with the respective vector alone. (A) Cdc25C cDNA transfected C-33 cells were cultured in regular medium and the cell number was then counted after 2 days. (B) Cdc25C shRNA-transfected C-33 cells were steroid starved for 48 hr in SR medium, and then treated with or without 10 nM DHT for 2 days. (C) Cdc25C shRNA transfected PC-3 cells were cultured in regular medium for 2 days, and the cell number was counted. The ratio of cell proliferation was calculated by normalizing the experimental cell number to that of control cells transfected with vector alone, respectively. Total cell lysate proteins were analyzed for Cdc25A, Cdc25B, Cdc25C, Cyclin B1 and/or Cyclin D1 proteins. β-actin was used as a loading control. The ratios of Cdc25C, Cyclin B1 and CyclinD1 protein levels to β-actin were calculated after semi-quantification by densitometric analyses on films with different exposure time periods. *p<0.05, n = 2×3; Bar, standard deviation.

We determined the role of Cdc25C in the growth of PCa cells. C-33 cells that have low levels of endogenous Cdc25C protein with a slow growth rate were transiently transfected with Cdc25C cDNA in viral DNA vector and cell growth was analyzed by counting cell numbers. Fig. 4A showed that the growth of Cdc25C cDNA-transfected C-33 cells was increased by about 80% in average (*p*<0.05). Western blot analyses validated that Cdc25C and Cyclin B1, but not Cyclin D1 protein levels, were elevated in Cdc25C cDNA-transfected cells, approximately 9- and 4-fold of that in control cells transfected with vector alone semi-quantified by densitometric analyses followed by normalizing to control cells (Fig. 4A, right panel).

To validate the role of Cdc25C in up-regulating PCa cell growth, we analyzed the effect of down-regulation of Cdc25C protein by shRNA. Transient knock-down of Cdc25C protein by shRNA in AS LNCaP C-33 in SR condition (Fig. 4B, left panel, column #3 *vs*. #1) and AI PC-3 cells in regular medium (Fig. 4C, left panel) resulted in approximately an average of 35% decrease of cell proliferation (*p*<0.05). Western blot analyses revealed that Cdc25C proteins were mostly reduced in shRNA-transfected C-33 and PC-3 cells (Fig. 4B, right panel, lane #3 *vs*. #1 & Fig. 4C, right panel). In those cells, Cdc25A and Cdc25B protein levels were not changed, indicating the specificity of the shRNA to Cdc25C (Figs. 4B and 4C). In Cdc25C knock-down cells, Cyclin B1 protein levels were concurrently decreased by 60% in C-33 cells in SR condition (Fig. 4B, right panel, lane #3 *vs*. #1) and over 90% in PC-3 cells (Fig. 4C, right panel). Interestingly, the Cyclin D1 protein level was not much changed in either Cdc25C cDNA-transfected or shRNA-transfected C-33 cells (Figs. 4A & 4B) and was reduced by about 65% in PC-3 cells (Fig. 4C).

We further determined the effect of the knockdown of Cdc25C on DHT-stimulated C-33 cell proliferation. As shown in Fig. 4B (left panel, column #4 *vs*. #3), androgens could not significantly stimulate the growth of Cdc25C-shRNA transfected C-33 cells, despite the fact that DHT partially restored both Cdc25C and Cyclin B1 protein levels (Fig. 4B, right panel, lane #4 *vs*. #3). In comparison, DHT significantly increased the growth of control cells transfected with vector alone by over 40% of the mean of cell number (Fig. 4B, left panel, column #2 *vs*. #1, *p<0.05). The data collectively support the notion that Cdc25C protein plays a critical role in regulating the basal as well as the androgen-stimulated proliferation of PCa cells.

### Effects of *de novo* biosynthesis inhibitors on Cdc25C protein level by androgens

**Figure 5:**
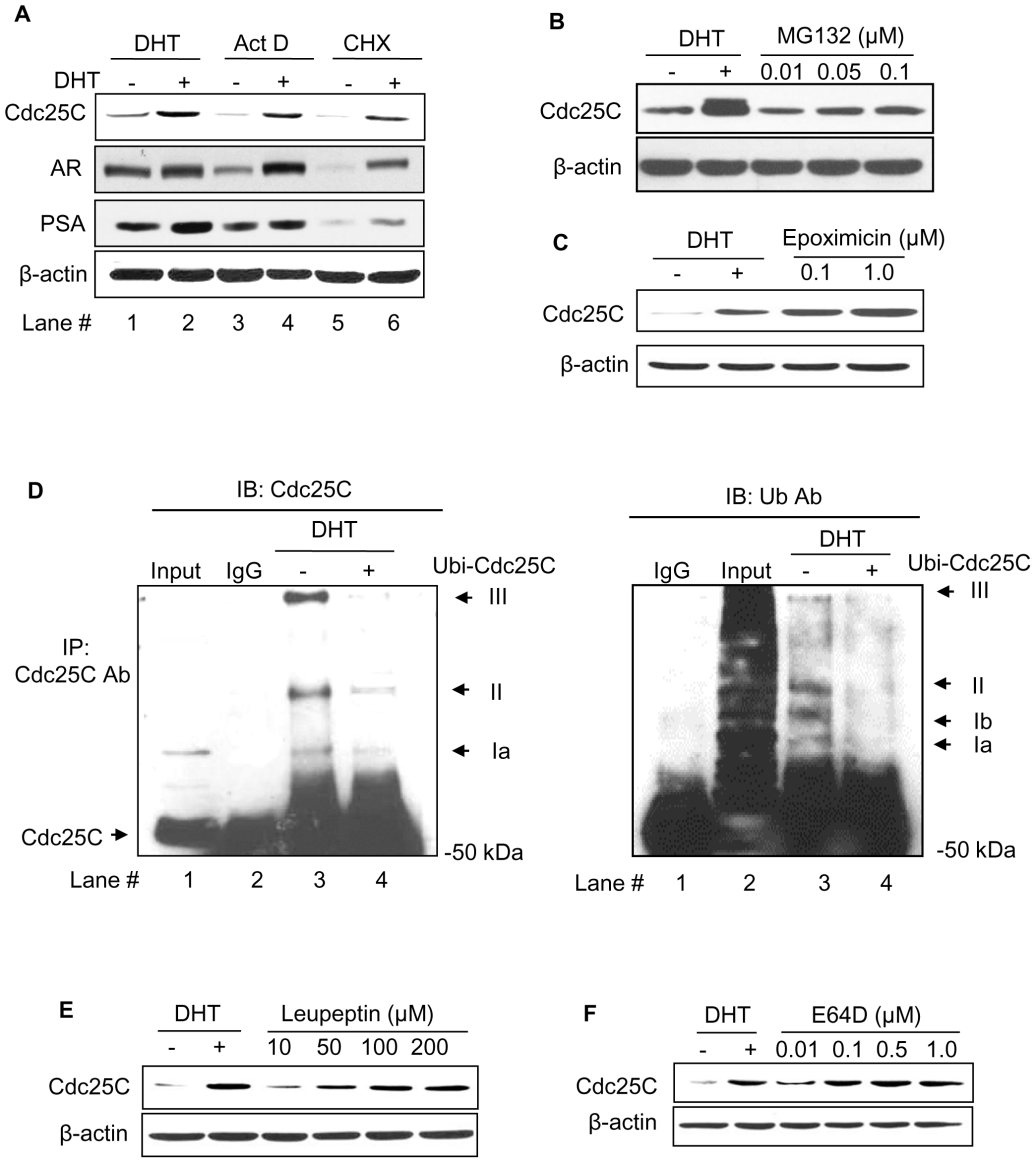
Effects of inhibition of protein biosynthesis and degradation pathways on Cdc25C protein levels. (A) Effects of *de novo* biosynthesis inhibitors on Cdc25C protein levels by androgens. LNCaP C-33 cells were plated at a density of 1.6×10^4^ cells/cm^2^ for 3 days in regular medium. Cells were steroid starved for 48 hr in a SR medium, and then treated with 5 µg/mL Actinomycin D (Act D) or 10 µg/mL cycloheximide (CHX) for 24 hr alone or 10 nM DHT was added 30 min post treatment with Act D and CHX. Total cell lysate proteins were analyzed for Cdc25C, AR and PSA protein level. β-actin was analyzed as a loading control. (B & C) Effects of inhibitors of proteasomal degradation pathway on Cdc25C protein level. LNCaP C-33 cells were seeded in regular medium for 72 hr, steroid starved for 48 hr and the cells were treated with or without 10 nM DHT or different concentrations of proteasomal inhibitors (B) MG132 (0.01, 0.05 and 0.1 µM) and (C) Epoximicin (0.1 and 1.0 M). Cells were harvested and analyzed for Cdc25C protein levels. β-actin was analyzed serving as a loading control. (D) Effect of androgens on the ubiquitination of Cdc25C protein. Steroid-starved LNCaP C-33 cells were treated with 10 nM DHT or solvent alone for 48 hr. An aliquot of total cellular lysate proteins was immunoprecipitated (IP) by reacting with anti-Cdc25C Ab or normal IgG and followed by Protein A-Sepharose beads. The immune complexes were analyzed by immunoblotting (IB) with anti-Cdc25C Ab (left panel) or anti-ubiquitin Ab (right panel). The positions of ubiquitinated Cdc25C protein (Ubi-Cdc25C) are indicated by arrows; while an additional band was detected by anti-ubiquitin Ab (Complex Ib). (E & F) Effects of inhibitors of lysosomal degradation pathway on Cdc25C protein levels. LNCaP C-33 cells were seeded as described in B & C, after steroid starvation, cells were treated with or without 10 nM DHT or different concentrations of lysosomal protease inhibitors. (E) Leupeptin (10 to 200 µM) and (F) E64D (0.01 to 1.0 µM) for 48 hr. Cells were harvested and analyzed for Cdc25C protein levels. β-actin was analyzed as a loading control.

We investigated the mechanism that androgens upregulate the Cdc25C protein level by analyzing if DHT increases Cdc25C protein biosynthesis. In LNCaP C-33 cells, in the absence of androgens, the basal Cdc25C protein level was further reduced by Act D and CHX treatments, inhibitors of *de novo* RNA synthesis and *de novo* protein synthesis, respectively (Fig. 5A, lanes #3 & #5 vs. #1). In the presence of DHT, Act D and CHX only had a partial effect on decreasing DHT-induced Cdc25C protein levels in these cells (Fig. 5A, lane #4 & #6 *vs*. #2). As controls, Act D only partially blocked DHT effects on AR and PSA protein levels (Fig. 5A, lane #4); while CHX can effectively block DHT-induced PSA, an androgen-regulated protein (Fig. 5A, lane #6). Thus, the data indicated that in DHT-treated cells, the elevation of Cdc25C protein level could not be explained by *de novo* biosynthesis alone.

### Effect of inhibitors of proteasomal and lysosomal degradation pathways on Cdc25C protein levels

We investigated the involvement of protein degradation pathways in up-regulating Cdc25C protein by DHT. Cdc25C protein was analyzed in C-33 cells treated with proteasomal inhibitors MG 132 (0.01, 0.05 and 0.1 µM) and epoximicin (0.1 and 1.0 µM). DHT effect on Cdc25C protein level was used as the positive control. As shown in Fig. 5B, in MG132-treated C-33 cells, Cdc25C protein level was elevated, following a dose-response fashion, although to a lesser degree than DHT effect. Epoximicin treatments also caused an increase of Cdc25C protein level with a more potent effect than DHT, following a dosage-response fashion (Fig. 5C). Interestingly, in DHT-treated cells, an additional Cdc25C band with a slightly slower mobility appeared, which is possibly due to phosphorylation (Fig. 5B). The data indicate that DHT up-regulation of the Cdc25C protein level could be in part via inhibiting the proteasomal degradation pathway.

Since ubiquitination signals proteasomal degradation, we investigated DHT effect on the ubiquitination of Cdc25C protein in DHT-treated LNCaP C-33 cells *vs*. control cells treated with the solvent alone. The total cell lysate proteins were immunoprecipitated with anti-Cdc25C Ab, and the immunocomplexes were analyzed by immunoblotting with anti-Cdc25C Ab (Fig. 5D, left panel) and anti-ubiquitin Ab (Fig. 5D, right panel), respectively. As shown in Fig. 5D (left panel, lane #3), in the absence of DHT, Cdc25C protein was highly ubiquitinated as indicated by the appearance of high molecular mass complexes by anti-Cdc25C Ab. The ubiquitinated Cdc25C protein complexes were greatly decreased in DHT-treated LNCaP C-33 cells (Fig. 5D, left panel, lane #4 *vs*. #3). Similarly, the ubiquitinated proteins by anti-ubiquitin Ab were decreased in DHT-treated C-33 cells (Fig. 5D, right panel, lane #4 *vs*. #3) despite an additional band was detected (Complex Ib in Fig. 5D, right panel, lane #3). Together these results support the notion that androgens reduce the degradation of Cdc25C in part by inhibiting its ubiquitination and elevated Cdc25C protein levels correlate with increased cell proliferation.

We also analyzed the effects of lysosomal protease inhibitors leupeptin (10 to 200 µM) and E64D (0.01 to 1.0 µM) on Cdc25C protein levels. Unexpectedly, the Cdc25C protein level was elevated in leupeptin-treated cells in a dose-dependent manner (Fig. 5E). Consistently, E64D, another lysosomal protease inhibitor, also elevated Cdc25C protein levels in a dosage-dependent manner (Fig. 5F). The data thus indicate that DHT-increased Cdc25C protein level is at least in part via the inhibition of its degradation pathways, possibly including both proteasomal and lysosomal pathways.

## Discussion

Cdc25 phosphatases play a critical role at the checkpoint for regulating cell cycle progression by dephosphorylating cyclin-dependent kinases. Aberrant expression of Cdc25C can lead to abnormal cell cycle progression, tumor initiation and progression. For example, increased expression of Cdc25C has been reported in a fraction of colon cancer cells [45], endometrial cancer [46] and prostate carcinomas [25]. In prostate carcinomas, Cdc25C and its spliced forms are upregulated at the mRNA and protein level [25]. However, the molecular mechanism of its upregulation in PCa cells remains an enigma. In this communication, our data clearly show for the first time that Cdc25C protein level is up-regulated by androgens in AS PCa cells, at least in part by decreasing its ubiquitination and degradation. In those cells, Cdc25C protein levels correlate with the basal as well as the androgen-stimulated cell proliferation. Furthermore, Cdc25C protein is elevated in at least a subfraction of AI PCa cells in a SR condition, and elevated expression of Cdc25C promotes PCa cell proliferation, which correlates with elevated Cyclin B1 protein level. Our data provide a mechanistic explanation of elevated Cdc25C protein levels on clinical prostate carcinomas and its role in PCa cell proliferation. Our results can have clinical significance toward advanced PCa therapy including castration-resistant PCa.

In regular culture condition which exhibits androgenic activity [47], the endogenous Cdc25C protein level, but not Cdc25A or B, is about 20% lower in AS LNCaP C-33 cells which have a slow growth rate and low tumorigenicity, than in AI LNCaP C-81 cells which grow rapidly and are highly tumorigenic, mimicking the advanced stages of PCa (Fig. 1) [26], [28], [41]. In SR condition, among three Cdc25 family members, Cdc25C protein exhibits the most sensitivity, and its protein level is greatly decreased in AS C-33 cells in reference to that in AI C-81 cells (Figs. 1 & 2). In the same SR-cultured C-33 cells, Cdc25A and Cdc25B proteins are decreased by only approximately 50% in comparison to the respective protein level in C-81 cells (Fig. 1). Interestingly, Cdc25B protein is shown to have over-expression in 97% of human PCa archival specimens, significantly higher than in non-cancerous prostate specimens, by immunohistochemistry staining and the protein level correlates with Gleason scores [48]. In the same study, Cdc25B is shown to function as a coactivator of AR in a hormone-dependent manner in LNCaP cells [48]. Despite that, the function of Cdc25B to AR is independent of its cell cycle function; Cdc25B is proposed to play a role in human prostate carcinogenesis [48]. It should be noted that Cdc25B is also shown to be a cofactor of estrogen receptor [49], [50]. While Cdc25B protein is expressed in all PCa cells examined in our experiments, our analyses on the expression profile of Cdc25 in cells cultured in both regular medium and SR condition (Fig. 1) and in the presence of androgens (Fig. 2A & 2B) show that androgens only have up-to 2-fold effect on Cdc25B protein level. It is possible that a 2-fold change in Cdc25B protein is sufficient for regulating its biological function. It is also possible that Cdc25B protein level is not directly regulated by androgens despite that it can function as an AR cofactor in the presence of androgens. Alternatively, in the PCa cells that were used in our experiments, the regulation of Cdc25B protein by androgens is altered. Further experiments with AS normal prostate epithelia are required to address this possibility. Nevertheless, our data indicate that Cdc25B and Cdc25C proteins are differentially regulated by androgens in PCa cells.

Importantly, decreased Cdc25C protein levels by shRNA results in the growth down-regulation of PCa cells in which Cdc25A and B protein levels are remained the same as in control cells (Fig. 4B & 4C). The data demonstrated the specificity of shRNA to Cdc25C. The data also indicate that Cdc25A and B exhibit unique biological activities in PCa cells, which are not over-lapping with that of Cdc25C and unable to cover the loss of Cdc25C function in promoting the growth of Cdc25C-knockdown cells. Interestingly, AS MDA PCa2b cells do not express Cdc25C protein with low Cyclin B1 level and exhibit a very slow growth rate even in the presence of 20% FBS, while AI DU 145 cells express Cdc25C and high Cyclin B1 level with rapid cell growth (data not shown). Furthermore, in Cdc25C cDNA or shRNA-transfected C-33 cells, Cyclin D1 level essentially remained the same (Fig. 4A & 4B) despite the fact that in PC-3 cells, Cyclin D1 is decreased by about 65% (Fig. 4C). Thus, Cdc25C signaling in up-regulating PCa cell growth is primarily transmitting through Cyclin B1, but not Cyclin D1.

Our data clearly demonstrate that in AS C-33 cells, DHT induces the elevation of Cdc25C protein level following a dose- (data not shown) and a kinetic-dependent manner (Fig. 2A). Nevertheless, in AI C-81 cells, 10 nM DHT exhibit an effect on Cdc25C protein levels by up-to 2-fold (Fig. 2A). Importantly, AR is required in this mode of regulation in AS C-33 cells (Fig. 2B & 2C). Evidently, when LNCaP C-33 and VCaP cells were concurrently treated with Casodex, an AR blocker used clinically in androgen deprivation therapy, DHT is unable to down-regulate cPAcP (Fig. 2B) or up-regulate the protein level of Cdc25C, Cyclin B1 or Cyclin D1 (Fig. 2B & 2C). In androgen-treated cells, cPAcP is down-regulated, which correlates with ErbB-2 and ERK/MAP kinases activation for promoting PCa cell proliferation (Fig. 2) [26], [27], [51]. In parallel, ERK-MAP kinases are shown to be at least in part involved in regulating Cdc25C during mitotic induction in ovarian cancer A2780 cells [52]. Furthermore, altered expression of Cdc25C protein, by cDNA and shRNA transfection, results in corresponding PCa cell growth regulation (Fig. 4). Thus, in DHT-treated cells, DHT interacts with AR for down-regulating cPAcP, a negative growth regulator, and up-regulating Cdc25C, a positive growth regulator; together, key components of the cell cycle including Cyclin B1 are up-regulated, resulting in promoting PCa cell proliferation.

Both DHT and EGF function as critical factors in up-regulating PCa cell proliferation, correlating with decreased cPAcP activity, a negative growth regulator of PCa cells by functioning as an authentic PTP (Fig. 2D) [29], [44] and activated ErbB-2 tyrosine phosphorylation signaling (Fig. 2D) [28], [51]. In fact, DHT-stimulated cell proliferation is in part mediated via ErbB-2 signaling pathway [28], [51]. Unexpectedly, only DHT, but not EGF, can significantly increase Cdc25C protein level in PCa cells, correlating with cell proliferation (Figs. 2D & 3). Interestingly, we observed that DHT, but not EGF, can increase a subfraction of tartrate-insensitive phosphatase activity [44]. Further experiments should clarify if Cdc25C represents the tartrate-insensitive phosphatase activity in androgen-stimulated PCa cells, which may have an important impact on PCa therapy [40], [44]. Additionally, while there could be other explanations, the data imply that DHT and EGF exhibit distinct signal pathways to stimulate PCa cell proliferation in addition to their cross-talks.

Unexpectedly, we observed broad peaks with the lack of G2 phase by fluorocytometry analyses (Fig. 3). Since those cells were originally purchased from ATCC and carefully maintained in our lab for establishing the PCa cell progression model (26,41), the broad peak may indicate the microheterogeneity nature of cell culture, including the heterogeneity of aneuploidy in those cells. Further, the lack of G2 can be due to the delay of S-phase or the premature mitotic exit. In parallel, it is commonly known that activation of Cdc25C triggers entry into mitosis and suppress G2/M checkpoint. Over-activation of Cdc25C may override the G2/M checkpoint and accelerates mitotic entry without proper pre-M phase activity, for example, DNA repair. This can lead to mitotic catastrophe because the regulation of mitotic exit also involves in the inactivation of Cdc25C. Over-activation of Cdc25C can lead to prolonged mitosis, which thus induces mitotic catastrophe. Nevertheless, further investigation is required to address androgen vs. EGF effect on Cdc25C protein involving in PCa cell proliferation.

Due to the importance of Cdc25C in regulating DHT-stimulated PCa cell growth, we elucidate the mechanism that DHT treatment increases Cdc25C protein. Our results showed that in the presence of a *de novo* protein biosynthesis inhibitor alone in the absence of DHT, the Cdc25C protein level was even lower than control cells (Fig. 5A, lane #3 & 5 vs. #1); while, an elevated level was observed when these inhibitors were used with DHT (Fig. 5A, lane #4 & 6 vs. #1). Thus, upregulation of Cdc25C could not be explained solely by that DHT up-regulates Cdc25C expression at the transcriptional level. In parallel, it has been shown that p53 protein represses Cdc25C expression by directly binding to the promoter of Cdc25C [53]. Nevertheless, our data revealed that p53 is not involved in DHT-upregulating Cdc25C protein in spite of LNCaP expressing the wild type of p53 protein. Evidently, the p53 protein level is not significantly changed in DHT-treated cells [54], [55] and DHT-induced elevation of Cdc25C protein is only partially sensitive to protein *de novo* biosynthesis inhibitors (Fig. 5A). Therefore, Cdc25C regulation by androgens, at least in PCa cells, follows a distinct mechanism to increase its protein level.

Our data show that the Cdc25C protein level is elevated when LNCaP cells are treated with proteasomal degradation pathway inhibitors, which is consistent with previous reports (Figs. 5B & 5C) [56], [57]. It is further supported by IP that androgens decrease the ubiquitination of Cdc25C protein (Fig. 5D). Unexpectedly, our results further revealed that the Cdc25C protein level is also elevated in C-33 cells treated by inhibitors toward the lysosomal protein degradation pathway (Figs. 5E & 5F). The data may indicate that mono- and poly-ubiquitinated proteins follow different routes for degradation [58]. Our data also indicate that androgen-regulating protein stability can be via different degradation pathways [54]. In summary, our data clearly show that in androgen-treated PCa cells, inhibition of Cdc25C protein degradation results in its elevated level. Our data further reveal for the first time that Cdc25C protein can be degraded via both proteasomal and lysosomal protein degradation pathways.

In summary, our data show for the first time that androgens stimulate cell growth at least in part through up-regulating Cdc25C protein by inhibiting its degradation and this promoting the cell cycle. In PCa cells, Cdc25C, but not Cdc25A or B, plays a critical role in cell growth regulation. In most of AI PCa cells, Cdc25C protein level is elevated. In the case of advanced PCa where cells can produce their own androgens [59] or in AI PCa cells that do not express AR, it can be beneficial to specifically inhibit Cdc25C for advanced castration-resistant PCa therapy. Our data provide new approaches for the development of novel therapeutics toward advanced stages of prostate cancer.

## References

[1] Satyanarayana A, Kaldis P (2009) Mammalian cell-cycle regulation: several Cdks, numerous cyclins and diverse compensatory mechanisms. Oncogene 28: 2925–2939. 19561645 10.1038/onc.2009.170

[2] Malumbres M, Barbacid M (2009) Cell cycle, CDKs and cancer: a changing paradigm. Nat Rev Cancer 9: 153–166. 19238148 10.1038/nrc2602

[3] Boutros R, Lobjois V, Ducommun B (2007) CDC25 phosphatases in cancer cells: key players? Good targets? Nat Rev Cancer 7: 495–507. 17568790 10.1038/nrc2169

[4] Boutros R, Dozier C, Ducommun B (2006) The when and wheres of CDC25 phosphatases. Curr Opin Cell Biol 18: 185–1891. 16488126 10.1016/j.ceb.2006.02.003

[5] Galaktionov K, Beach D (1991) Specific activation of cdc25 tyrosine phosphatases by B-type cyclins: evidence for multiple roles of mitotic cyclins. Cell 67: 1181–1194. 1836978 10.1016/0092-8674(91)90294-9

[6] Nagata A, Igarashi M, Jinno S, Suto K, Okayama H (1991) An additional homolog of the fission yeast cdc25+ gene occurs in humans and is highly expressed in some cancer cells. New Biol 3: 959–968. 1662986

[7] Ray D, Terao Y, Nimbalkar D, Hirai H, Osmundson EC, et al (2007) Hemizygous disruption of Cdc25A inhibits cellular transformation and mammary tumorigenesis in mice. Cancer Res 67: 6605–6611. 17638870 10.1158/0008-5472.CAN-06-4815

[8] Lincoln AJ, Wickramasinghe D, Stein P, Schultz RM, Palko ME, et al (2002) Cdc25b phosphatase is required for resumption of meiosis during oocyte maturation. Nat Genet 30: 446–449. 11912493 10.1038/ng856

[9] Chen MS, Hurov J, White LS, Woodford-Thomas T, Piwnica-Worms H (2001) Absence of apparent phenotype in mice lacking Cdc25C protein phosphatase. Mol Cell Biol 21: 3853–3861. 11359894 10.1128/MCB.21.12.3853-3861.2001 PMC87049

[10] Turowski P, Franckhauser C, Morris MC, Vaglio P, Fernandez A, et al (2003) Functional cdc25C dual-specificity phosphatase is required for S-phase entry in human cells. Mol Biol Cell 14: 2984–2998. 12857880 10.1091/mbc.E02-08-0515 PMC165692

[11] Kiyokawa H, Ray D (2008) In vivo roles of CDC25 phosphatases: biological insight into the anti-cancer therapeutic targets. Anticancer Agents Med Chem 8: 832–836. 19075565 10.2174/187152008786847693 PMC2753225

[12] Bonnet J, Mayonove P, Morris MC (2008) Differential phosphorylation of Cdc25C phosphatase in mitosis. Biochem Biophys Res Commun 370: 483–488. 18384749 10.1016/j.bbrc.2008.03.117

[13] Busch C, Barton O, Morgenstern E, Götz C, Günther J, et al (2007) The G2/M checkpoint phosphatase cdc25C is located within centrosomes. Int J Biochem Cell Biol 39: 1707–1713. 17548228 10.1016/j.biocel.2007.04.022

[14] Hoffmann I (2000) The role of cdc25 phosphatases in cell cycle checkpoints. Protoplasma 211: 8–11.

[15] Kawabe T, Suganuma M, Ando T, Kimura M, Hori H, et al (2002) Cdc25C interacts with PCNA at G2/M transition. Oncogene 21: 1717–1726. 11896603 10.1038/sj.onc.1205229

[16] Perdiguero E, Nebreda AR (2004) Regulation of Cdc25C activity during the meiotic G2/M transition. Cell Cycle 3: 733–737. 15136768

[17] Wang R, He G, Nelman-Gonzalez M, Ashorn CL, Gallick GE, et al (2007) Regulation of Cdc25C by ERK-MAP kinases during the G2/M transition. Cell 128: 1119–1132. 17382881 10.1016/j.cell.2006.11.053

[18] Matsuoka S, Huang M, Elledge SJ (1998) Linkage of ATM to cell cycle regulation by the Chk2 protein kinase. Science 282: 1893–1897. 9836640 10.1126/science.282.5395.1893

[19] Peng CY, Graves PR, Thoma RS, Wu Z, Shaw AS, et al (1997) Mitotic and G2 checkpoint control: regulation of 14-3-3 protein binding by phosphorylation of Cdc25C on serine-216. Science 277: 1501–1505. 9278512 10.1126/science.277.5331.1501

[20] Sanchez Y, Wong C, Thoma RS, Richman R, Wu Z, et al (1997) Conservation of the Chk1 checkpoint pathway in mammals: linkage of DNA damage to Cdk regulation through Cdc25. Science 277: 1497–1501. 9278511 10.1126/science.277.5331.1497

[21] Graves PR, Yu L, Schwarz JK, Gales J, Sausville EA, et al (2000) The Chk1 protein kinase and the Cdc25C regulatory pathways are targets of the anticancer agent UCN-01. J Biol Chem 275: 5600–5605. 10681541 10.1074/jbc.275.8.5600

[22] Bartek J, Lukas J (2003) Chk1 and Chk2 kinases in checkpoint control and cancer. Cancer Cell 3: 421–429. 12781359 10.1016/s1535-6108(03)00110-7

[23] Zeng Y, Forbes KC, Wu Z, Moreno S, Piwnica-Worms H, et al (1998) Replication checkpoint requires phosphorylation of the phosphatase Cdc25 by Cds1 or Chk1. Nature 395: 507–510. 9774107 10.1038/26766

[24] Nigg EA (2001) Mitotic kinases as regulators of cell division and its checkpoints. Nat Rev Mol Cell Biol 2: 21–32. 11413462 10.1038/35048096

[25] Ozen M, Ittmann M (2005) Increased expression and activity of CDC25CPhosphatase and an alternatively spliced variant in prostate cancer. Clin Cancer Res 11: 4701–4706. 16000564 10.1158/1078-0432.CCR-04-2551

[26] Lin MF, Meng TC, Rao PS, Chang C, Schonthal AH, et al (1998) Expression of human prostatic acid phosphatase correlates with androgen-stimulated cell proliferation in prostate cancer cell lines. J Biol Chem 273: 5939–5947. 9488733 10.1074/jbc.273.10.5939

[27] Chuang TD, Chen SJ, Lin FF, Veeramani S, Kumar S, et al (2010) Human prostatic acid phosphatase, an authentic tyrosine phosphatase, dephosphorylates ErbB-2 and regulates prostate cancer cell growth. J Biol Chem 285: 23598–23606. 20498373 10.1074/jbc.M109.098301 PMC2911278

[28] Lin MF, Lee MS, Zhou XW, Andressen JC, Meng TC, et al (2001) Decreased expression of cellular prostatic acid phosphatase increases tumorigenicity of human prostate cancer cells. J Urol 166: 1943–1950. 11586265

[29] Lin MF, DaVolio J, Garcia-Arenas R (1992) Expression of human prostatic acid phosphatase activity and the growth of prostate carcinoma cells. Cancer Res 52: 4600–4607. 1380886

[30] Veeramani S, Yuan TC, Chen SJ, Lin FF, Petersen JE, et al (2005) Cellular prostatic acid phosphatase: a protein tyrosine phosphatase involved in androgen-independent proliferation of prostate cancer. Endocr Relat Cancer 12: 805–822. 16322323 10.1677/erc.1.00950

[31] Meng TC, Lin MF (1998) Tyrosine phosphorylation of c-ErbB-2 is regulated by the cellular form of prostatic acid phosphatase in human prostate cancer cells. J Biol Chem 273: 22096–22104. 9705354 10.1074/jbc.273.34.22096

[32] Lee MS, Igawa T, Lin MF (2004) Tyrosine-317 of p52(Shc) mediates androgen-stimulated proliferation signals in human prostate cancer cells. Oncogene 23: 3048–3058. 14990987 10.1038/sj.onc.1207451

[33] Lee MS, Igawa T, Chen SJ, Van Bemmel D, Lin JS, et al (2004) p66Shc protein is upregulated by steroid hormones in hormone-sensitive cancer cells and in primary prostate carcinomas. Int J Cancer 108: 672–678. 14696093 10.1002/ijc.11621

[34] Horoszewicz JS, Leong SS, Kawinski E, Karr JP, Rosenthal H, et al (1983) LNCaP model of human prostatic carcinoma. Cancer Res 43: 1809–1818. 6831420

[35] Navone NM, Olive M, Ozen M, Davis R, Troncoso P, et al (1997) Establishment of two human prostate cancer cell lines derived from a single bone metastasis. Clin Cancer Res 3: 2493–2500. 9815652

[36] Kaighn ME, Narayan KS, Ohnuki Y, Lechner JF, Jones LW (1979) Establishment and characterization of a human prostatic carcinoma cell line (PC-3). Invest Urol 17: 16–23. 447482

[37] Stone KR, Mickey DD, Wunderli H, Mickey GH, Paulson DF (1978) Isolation of a human prostate carcinoma cell line (DU 145). Int J Cancer 21: 274–281. 631930 10.1002/ijc.2910210305

[38] Korenchuk S, Lehr JE, MClean L, Lee YG, Whitney S, et al (2001) VCaP, a cell-based model system of human prostate cancer. In Vivo 15: 163–168. 11317522

[39] Chen SJ, Karan D, Johansson SL, Lin FF, Zeckser J, et al (2007) Prostate-derived factor as a paracrine and autocrine factor for the proliferation of androgen receptor-positive human prostate cancer cells. Prostate 67: 557–571. 17221842 10.1002/pros.20551

[40] Veeramani S, Chou YW, Lin FC, Muniyan S, Lin FF, et al (2012) Reactive oxygen species induced by p66Shc longevity protein mediate nongenomic androgen action via tyrosine phosphorylation signaling to enhance tumorigenicity of prostate cancer cells. Free Radic Biol Med 53: 95–108. 22561705 10.1016/j.freeradbiomed.2012.03.024 PMC3384717

[41] Igawa T, Lin FF, Lee MS, Karan D, Batra SK, et al (2002) Establishment and characterization of androgen-independent human prostate cancer LNCaP cell model. Prostate 50: 222–235. 11870800 10.1002/pros.10054

[42] Telford WG, King LE, Fraker PJ (1991) Evaluation of glucocorticoid-induced DNA fragmentation in mouse thymocytes by flow cytometry. Cell Prolif 24: 447–459. 1657218 10.1111/j.1365-2184.1991.tb01173.x

[43] Chou YW, Chaturvedi NK, Ouyang S, Lin FF, Kaushik D, et al (2011) Histone deacetylase inhibitor valproic acid suppresses the growth and increases the androgen responsiveness of prostate cancer cells. Cancer Lett 311: 177–186. 21862211 10.1016/j.canlet.2011.07.015 PMC3232184

[44] Lin MF, Lee CL, Clinton GM (1986) Tyrosyl kinase activity is inversely related to prostatic acid phosphatase activity in two human prostate carcinoma cell lines. Mol Cell Biol 6: 4753–4757. 3796616 10.1128/mcb.6.12.4753-4757.1986 PMC367263

[45] Hernández S, Bessa X, Beà S, Hernández L, Nadal A, et al (2001) Differential expression of cdc25 cell-cycle-activating phosphatases in human colorectal carcinoma. Lab Invest 81: 465–473. 11304565 10.1038/labinvest.3780254

[46] Tsuda H, Hashiguchi Y, Inoue T, Yamamoto K (2003) Alteration of G2 cell cycle regulators occurs during carcinogenesis of the endometrium. Oncology 65: 159–166. 12931023 10.1159/000072342

[47] Lin MF, Lee MS, Garcia-Arenas R, Lin FF (2000) Differential responsiveness of prostatic acid phosphatase and prostate-specific antigen mRNA to androgen in prostate cancer cells. Cell Biol Int 24: 681–689. 11023645 10.1006/cbir.2000.0433

[48] Ngan ES, Hashimoto Y, Ma ZQ, Tsai MJ, Tsai SY (2003) Overexpression of Cdc25B, an androgen receptor coactivator, in prostate cancer. Oncogene 22: 734–739. 12569365 10.1038/sj.onc.1206121

[49] Ma ZQ, Liu Z, Ngan ES, Tsai SY (2001) Cdc25B functions as a novel coactivator for the steroid receptors. Mol Cell Biol 21: 8056–8067. 11689696 10.1128/MCB.21.23.8056-8067.2001 PMC99972

[50] Wu W, Slomovitz BM, Celestino J, Chung L, Thornton A, et al (2003) Coordinate expression of Cdc25B and ER-alpha is frequent in low-grade endometrioid endometrial carcinoma but uncommon in high-grade endometrioid and nonendometrioid carcinomas. Cancer Res 63: 6195–6199. 14559803

[51] Meng TC, Lee MS, Lin MF (2000) Interaction between protein tyrosine phosphatase and protein tyrosine kinase is involved in androgen-promoted growth of human prostate cancer cells. Oncogene 19: 2664–2677. 10851066 10.1038/sj.onc.1203576

[52] Wang R, He G, Nelman-Gonzalez M, Ashorn CL, Gallick GE (2007) Regulation of Cdc25C by ERK-MAP kinases during the G2/M transition. Cell 128: 1119–1132. 17382881 10.1016/j.cell.2006.11.053

[53] St Clair S, Manfredi JJ (2006) The dual specificity phosphatase Cdc25C is a direct target for transcriptional repression by the tumor suppressor p53. Cell Cycle 5: 709–713. 16582636 10.4161/cc.5.7.2628

[54] Kumar S, Kumar S, Rajendran M, Alam SM, Lin FF, et al (2011) Steroids up-regulate p66Shc longevity protein in growth regulation by inhibiting its ubiquitination. PLoS One 6: e15942. 21264241 10.1371/journal.pone.0015942 PMC3021521

[55] Veeramani S, Yuan TC, Lin FF, Lin MF (2008) Mitochondrial redox signaling by p66Shc is involved in regulating androgenic growth stimulation of human prostate cancer cells. Oncogene 27: 5057–5068. 18504439 10.1038/onc.2008.143 PMC2776635

[56] Chen F, Zhang Z, Bower J, Lu Y, Leonard SS, et al (2002) Arsenite-induced Cdc25C degradation is through the KEN-box and ubiquitin-proteasome pathway. Proc Natl Acad Sci U S A 99: 1990–1995. 11842186 10.1073/pnas.032428899 PMC122307

[57] Eymin B, Claverie P, Salon C, Brambilla C, Brambilla E, et al (2006) p14ARF triggers G2 arrest through ERK-mediated Cdc25C phosphorylation, ubiquitination and proteasomal degradation. Cell Cycle 5: 759–765. 16582626 10.4161/cc.5.7.2625

[58] Haglund K, Sigismund S, Polo S, Szymkiewicz I, Di Fiore PP, et al (2003) Multiple monoubiquitination of RTKs is sufficient for their endocytosis and degradation. Nat Cell Biol 5: 461–466. 12717448 10.1038/ncb983

[59] Dillard PR, Lin MF, Khan SA (2008) Androgen-independent prostate cancer cells acquire the complete steroidogenic potential of synthesizing testosterone from cholesterol. Mol Cell Endocrinol 295: 115–120. 18782595 10.1016/j.mce.2008.08.013 PMC2802176

